# Effect of postoperative ultrasound-guided internal superior laryngeal nerve block on sore throat after intubation of double-lumen bronchial tube: a randomized controlled double-blind trial

**DOI:** 10.1186/s12871-022-01819-x

**Published:** 2022-09-01

**Authors:** Jingxian Wang, Bin Chai, Yujie Zhang, Lidong Zheng, Pengcheng Geng, Li Zhan

**Affiliations:** 1 Department of Anesthesiology, Lu’an Affiliated Hospital of Anhui Medical University, No.21 West Wanxi Road, Jinan District, Lu’an, 237000 Anhui China; 2 Department of Anesthesiology, Zhongshan Hospital Affiliated to Xiamen University, Xiamen, Fujian China

**Keywords:** Anesthesia, Intubation, Ultrasound, Postoperative sore throat, Nerve block

## Abstract

**Background:**

Postoperative sore throat (POST) is one of the main adverse postoperative outcome after tracheal intubation using double-lumen endobronchial tubes (DLTs). The aim of this study was to investigate the effectiveness and safety of ultrasound (US)-guided block of the internal branch of the superior laryngeal nerve (iSLN) for alleviating POST after intubation of DLTs.

**Methods:**

Patients undergoing thoracic surgery between August 2019 and August 2021 were randomized into two groups depending on whether they received US-guided iSLN block immediately after the operation. In the control group, the patients underwent a thoracic surgery under general anesthesia (GA) with DLTs without any special treatment, while the patients in the experimental group received US-guided iSLN block bilaterally with 2 ml of 0.25% ropivacaine on either side immediately after the operation. The primary outcome was the grading of sore throat at three-time points after the operation, i.e., immediate extubation, 2 h after extubation, and 24 h after extubation. Secondary outcomes included the rate of nausea and vomiting, hoarseness, dyspnea, and choking cough after swallowing saliva at 2 h after extubation.

**Results:**

The incidence and severity of sore throat were significantly lower in the experimental group than the control group at all time intervals (all *P* < 0.01). The rate of nausea and vomiting, hoarseness, dyspnea, and choking cough after swallow saliva at 2 h after extubation had no statistical difference (all *P* > 0.05).

**Conclusions:**

The use of US-guided iSLN block can be effectively and safely applied to relieve POST after intubation of DLTs on thoracic surgery.

**Trial registration:**

The study protocol was registered at the Chinese Clinical Trial Registry (http://www.chictr.org.cn, NO. ChiCTR2000032188, 22/04/2020).

## Background

Lung isolation facilitates surgical access during thoracotomy and video-imaged thoracic surgery. The double-lumen endobronchial tube is generally considered the gold standard for lung isolation. Intubation with double-lumen tubes, which have a larger external diameter than conventional single-lumen tubes, is especially likely to provoke sore throats, with a reported incidence of up to 90% [1]. Although POST symptoms may appear minor, POST can negatively affect patients’ recovery and satisfaction [2], thus making them uncomfortable and anxious during the postoperative period. Therefore, successfully relieving POST is an important issue requiring anesthesiologists’ attention. Many interventions have been proposed to decrease the incidence of POST after intubation, such as lubricating the endotracheal tube with water-soluble jelly, minimizing cuff pressure, extubating in deep anesthesia, use of intravenous lidocaine [3], inhalation of beclomethasone [4], and topical ketamine [5]. Seo et al demonstrated that thermal softening of DLTs before intubation could decrease the postoperative incidence of sore throat and vocal cord injuries [6].

The superior laryngeal nerve is a branch of the vagus nerve, which bifurcates into an internal and external branch, where the internal branch provides sensory innervation of the tongue, epiglottis, and the mucous membrane of the larynx down to the vocal cords [7]. In general, superior laryngeal nerve blocks with lidocaine have been reported to effectively anesthetize the larynx for airway manipulation and pain management [8, 9]. Ramkumar et al have reported that superior laryngeal nerve block as an adjuvant to general anesthesia can provide a better recovery profile of the patients and reduce the occurrence of postoperative cough, sore throat, and hoarseness of voice [10]. As is well known, due to the visualization of ultrasound, ultrasound-guided nerve block locks are more likely to be successful, they are less time-consuming, and have been associated with fewer complications [11].

As many researchers performed the block before surgery and also blocked the external branch of SLN [10], the aim of the present study was to investigate the effectiveness and safety of US-guided bilateral iSLN block for alleviating POST immediately after the operation.

## Methods

### Study design

This study was conducted after receiving approval from the local Research Ethics Committee (2019–003-01, April 10, 2019) and registered with the Chinese Clinical Trial Registry (http://www.chictr.org.cn, NO. ChiCTR2000032188, 22/04/2020). Written informed consent was obtained from all participants. All procedures were performed in accordance with the ethical standards laid down in the 1964 Declaration of Helsinki and its later amendments.

**Fig. 1:**
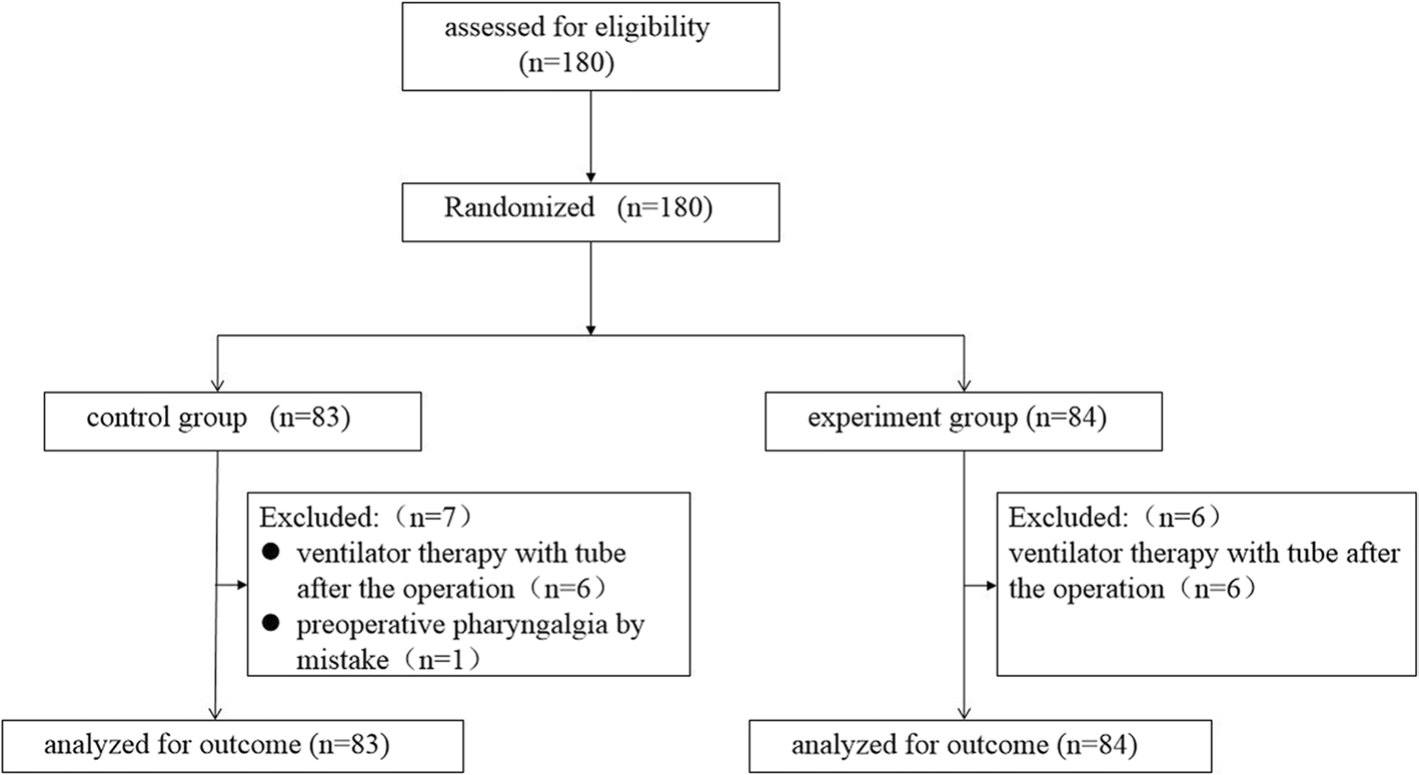
The flow of participants through the study

According to the pre-experimental results, A total of 180 adult patients (males and females) who were scheduled for thoracic surgery under general anesthesia with double-lumen bronchial intubation were recruited into the study. Inclusion criteria were: American Society of Anesthesiologists (ASA) I- III; scheduled to undergo thoracic surgery; age of 18–75 years; and provision of informed consent for participation in the study. Exclusion criteria were the following: patients who did not provide consent; with a pre-existing sore throat, hoarseness and upper respiratory tract infection, tracheal pathology including tracheostomy; a history of diabetes or psychosis disease and surgery on the thyroid, oral cavity, or pharynx; known or suspected allergy to ropivacaine; use of nonsteroidal anti-inflammatory drug medication within 24 hours, chronic opioid use, and known or suspected difficult airway (anesthesiologists with more than 5 years of clinical anesthesia experience encounter difficulties with mask ventilation (upper airway obstruction), or with tracheal intubation, or a combination of both). Patients who were supported by a tube ventilator after operation were excluded from the study. This experiment was conducted using a randomized, double-blind design. The SAS statistical software was used to generate a table of random numbers and group numbers in the ratio of 1:1. Each patient was assigned a random number in the order of surgery time and then grouped accordingly. (Fig. 1). Both patients and investigators observing the patients in the postoperative period and analyzing the data were blinded to group allocation.

The patients were divided into 2 following groups: the control group, in which patients underwent a thoracic surgery under general anesthesia with double-lumen bronchial intubation without any special treatment; and the experimental group, in which patients received US-guided iSLN block bilaterally with 2 ml of 0.25% ropivacaine on either side immediately after the operation along with GA to undergo thoracic surgery. All the patients presented to operation theatre on the day of surgery after overnight fasting for 8 hours and not drinking for 4 hours. Standard monitoring techniques including heart rate (HR), electrocardiogram (ECG), non-invasive blood pressure (NIBP), oxygen saturation (SpO_2_), bispectral index (BIS), end-tidal carbon dioxide partial pressure (ETCO_2_), and temperature were measured using multichannel monitors (Mindray T8, Mindray Company, Shen Zhen, CN). General anesthesia (GA) was induced with intravenous (i.v.) midazolam 0.02 mg/kg for anxiolysis, i.v. sufentanil 0.5μg/kg, i.v. etomidate 0.4 mg/kg and neuromuscular block was achieved with rocuronium (0.6–0.9 mg/kg). Double lumen endotracheal intubations were performed by trainees under the guidance of the registered (consultant) anesthesiologist or by the consultant anesthesiologist using a visual laryngoscope under muscle looseness monitoring (Guangzhou Weili Ark Technology Company, Veryark-TOF). All these trainees have completed a simulation-based training course in airway management and passed the practical examination on airway management. All the DLTs were adequately lubricated. The DLTs size was chosen based on the sex and height of the patients [12, 13]. Then, the DLTs position was correctly adjusted under fiberoptic bronchoscopy. Paravertebral block was used in all patients postoperatively.

**Fig. 2:**
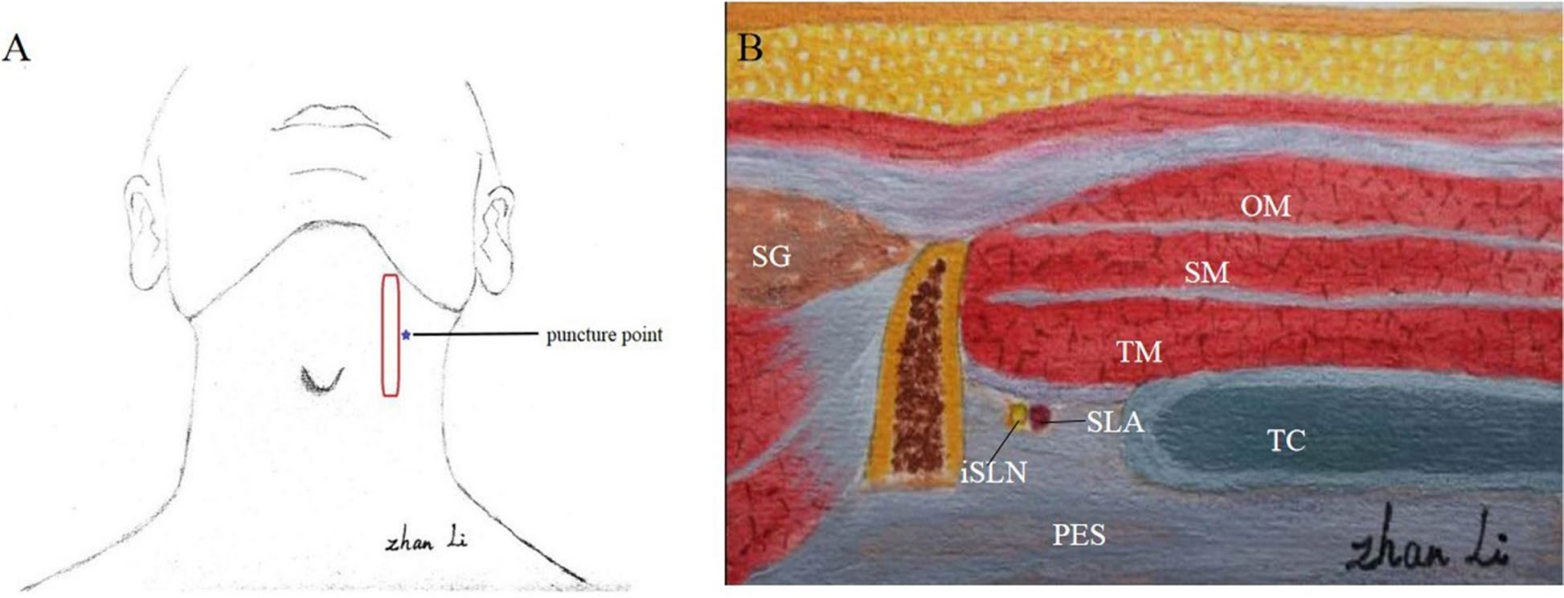
**A** Probe and puncture point. **B** Anatomy of the internal branch of the superior laryngeal nerve. OM, omohyoid muscle; SM, sternohyoid muscle; TM, thyrohyoid muscle; HB, hyoid bone; SLA, superior laryngeal artery; iSLN, internal branch of the superior laryngeal nerve; SG, submandibular gland; PES, pre-epiglottis space; TC: thyroid cartilage

**Fig. 3:**
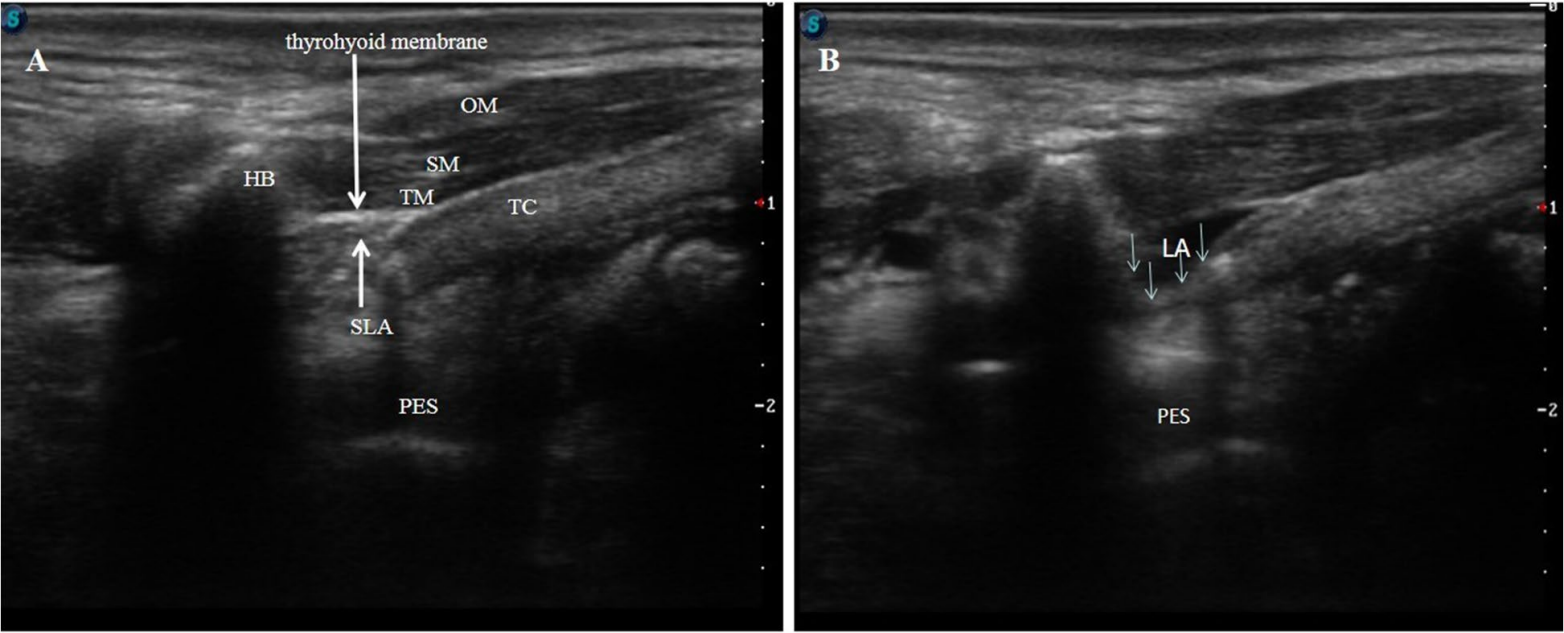
**A** Ultrasound images of a longitudinal iSLN. **B** Local anesthetic was injected into the superior laryngeal nerve space. OM, omohyoid muscle; SM, sternohyoid muscle; TM, thyrohyoid muscle; SLA, superior laryngeal artery; LA, local anesthetic; PES, pre-epiglottis space. TC: thyroid cartilage

In the experimental group, the patient was placed in the supine position with the neck extended, and a US-guided longitudinal iSLN block was performed immediately after surgery. A linear 4- to 12-MHz ultrasound probe (UMT-400; Mindray TE7, Shen Zhen, CN) was placed in the oblique parasagittal plane to obtain an image of the hyoid bone and the thyroid cartilage (Fig. 2A). The probe was kept parasagitally, and the following structures were identified anteriorly to posteriorly: omohyoid muscle, sternohyoid muscle, thyrohyoid muscle, thyrohyoid membrane, superior laryngeal artery (the artery is small and may not be visible), and pre-epiglottis space (Figs. 2B, 3A). After identifying the local anatomic structure, a needle was inserted out-plane from anterior to posterior. The muscles above the thyrohyoid membrane were penetrated and hydrodissected; local anesthetic was injected into the superior laryngeal nerve space (Fig. 3B). The superior laryngeal nerve space was located between the hyoid bone (cephalad) and the thyroid cartilage (caudal) and delimited between the thyrohyoid muscle, anteriorly, and the thyrohyoid membrane and the pre-epiglottis space, posteriorly [14]. All the steps were carried out under aseptic conditions.

### Measurement of outcomes

The primary indicator was the pain classification of postoperative sore throat. Postoperative sore throat was assessed by clinical observation and questioning the patient immediately after extubation and at 2 and 24 hours after extubation. An established scoring system was used: (1) 0 point; (2) 1 point (less than a common cold); (3) 2 point (similar to a common cold); and (4) 3 point (more than a common cold) [15].

The secondary indicators included the rate of nausea and vomiting, hoarseness, dyspnea, and choking cough after swallowing saliva at 2 h after extubation.

### Statistical analysis

Continuous quantitative normally distributed data were expressed as means and standard deviations (SD). Quantitative discrete data were expressed as median and range. Patients’ age, height, weight, and duration of anesthesia were compared between groups and statistically tested by analysis of two independent samples t-test. Between-group differences in patients’ sex were analyzed by Fisher’s exact test. Rank sum test was used for comparison of grade data; Mann-Whitney U test was used for between-group differences; Kruskal-Wallis H test was used for within-group differences. A *p-*value < 0.05 was considered statistically significant. All analyses were performed in SPSS (version 19.0; SPSS Inc., IBM, Chicago, IL, USA).

## Results

A total of 180 patients were included in the present study. Figure 1 presents the allocation process according to the Consolidated Standards of Reporting Trials. Twelve patients (6 from each group) were excluded from the analysis as they needed ventilator therapy with a tube after the operation. In addition, 1 patient from the experimental group withdrew due to erroneous preoperative pharyngalgia as follow-up after surgery showed that this patient had sore throat 2 months before surgery (Fig. 1).

Demographic data were similar between the two groups. There was no significant difference in age, sex, body weight, or height between the groups. In addition, the time of anesthesia did not significantly differ between the two groups (Table 1). The results showed no difference in DLT size between the two groups.

**Table 1:** Demographic data and anesthesia time by study group

group	n	Sex (M/F)	age (year)	height (cm)	weight (kg)	Anesthesia time (min)	Size of DLTs (37/35/32)
control	84	52/32	59.60 ± 11.330	165.43 ± 7.827	62.62 ± 10.930	193.00 ± 81.753	40/34/10
experiment	83	48/35	56.14 ± 14.494	164.38 ± 8.142	62.37 ± 9.538	172.30 ± 78.403	54/21/8
χ^2^ or t		0.288^a^	1.715	0.844	0.155	1.670	1.454
*P* value		0.591	0.088	0.400	0.877	0.097	0.146

Data are expressed as mean ± SD or numbers. There were no significant differences between groups

The total incidence of sore throat was significantly lower in the experimental group compared with the control group (*P* < 0.05) immediately as well as 2 and 24 hours after extubation (Table 2). None of the patients from the experimental group had moderate or severe (grades 2 and 3) sore throat immediately after extubation. However, in the control group, moderate and severe symptoms were reported in 11 patients for sore throat. In addition, none of the patients in the experimental group had a severe sore throat at 2 and 24 hours after extubation.

**Table 2:** Data are presented as numbers of patients who suffered POST at immediate extubation, 2 h, 24 h after extubation

Group	n	Immediate extubation				2 h after extubation				24 h after extubation			
		0	1	2	3	0	1	2	3	0	1	2	3
Control	84	51	22	9	2	44	27	11	2	45	31	5	3
Experiment	83	77	6	0	0	70	10	3	0	69	12	2	0
Ζ		−4.98				−4.462				−4.124			
*p*-value		0.000				0.000				0.000			

(1) none; (2) mild (less than a common cold); (3) moderate (similar to a common cold); and (4) severe (more than a common cold)

The incidence of nausea, vomiting, hoarseness, dyspnea, and choking cough after swallowing saliva at 2 h after extubation did not significantly differ between groups (Table 3).

**Table 3:** Data are presented as numbers of patients who suffered nausea and vomiting, hoarseness, dyspnea, and choking cough after swallow saliva at 2 h after extubation

Group	n	Choking cough after swallow	Hoarsenes	Dyspnea	Nausea and Vomiting
Control	84	1	3	2	8
Experiment	83	1	2	1	7
*P* value		0.99	0.66	0.57	0.81

## Discussion

Our data showed that the incidence of sore throat was lower in patients who received iSLN block immediately after extubation, 2 and 24 hours after extubation, thus suggesting that US-guided iSLN block could ameliorate the incidence of POST induced by double-lumen bronchial catheter intubation.

POST is a common complication after general anesthesia via tracheal intubation that may be distressing for the patient. Furthermore, using a DLT increases the incidence and intensity of postoperative sore throat compared with using a single-lumen tube [16]. As is well known, POST has no serious risk or great influence on the surgical prognosis, so it is often neglected by anesthesiologists and surgeons. Yet, we found that POST is one of the most adverse effects patients experience after surgery. POST associated with tracheal intubation can be influenced by many factors, such as gender, smoking, intubating conditions, size, and intra-cuff pressure of tubes, and type or duration of surgery [17]. In our study, there was no statistical difference in these risk factors. A number of methods for reducing POST have been used for the double-lumen endobronchial tubes. Numerous studies mainly focused on using drugs to prevent POST [18, 19]. Also, many other methods have also been found to prevent POST. One study reported that silicon DLTs led to a lower incidence of postoperative sore throat compared to the use of PVC DLTs [20]. Seo et al demonstrated that rotation of a DLT through 180° facilitated its passage through the glottis and reduced the incidence of postoperative sore throat [21]. Still, previous studies reported the inconsistent effect in relation to these techniques. The present study showed that the iSLN block produced better results without complications. It is well known that in recent years, anesthesiologists are increasingly using ultrasound techniques to perform nerve blocks. In our study, we demonstrated that ultrasound-guided superior laryngeal nerve blocks could ameliorate postoperative sore throat after double-lumen bronchial catheter intubation.

The greatest occurrence of POST was at 2–4 hours after extubation, which is consistent with previous reports [22]. Patients might not have been sufficiently awake, cooperative, or out of delirium to appropriately answer the POST questions at the time of immediate extubation point. In the present study, the POST frequency was 46.4% in the control group at 24 h postoperatively, and this incidence was consistent with previous reports [23]. Nevertheless, the US-guided iSLN block significantly decreased the incidence of POST.

As is well known, the sensory innervations of the larynx, a potent reflexogenic region, are provided by the internal branch of the superior laryngeal nerve. Numerous studies have used superior laryngeal nerve blocks for the cure of neurogenic cough [24, 25]. On the other hand, a bilateral block of this nerve has been commonly performed as a part of upper airway preparation for awake intubation [8, 26]. The present study showed that US-guided iSLN block helped in attenuating the incidence and severity of POST. We performed bilateral iSLN block under ultrasound guidance so as to increase the success rate of the block. Our results showed that the effect of relieving POST continued until 24 h after operation, but not after that. In our study, the local anesthetic was injected into the superior laryngeal nerve space, while the external branch of the superior laryngeal nerve and recurrent laryngeal nerve were not blocked. However, the incidence of choking cough after swallowing saliva, hoarseness, and dyspnea at 2 h after extubation revealed no significant difference between the two groups, thus confirming the safety of this method.

There are some limitations in the present study. First, no further observations were done beyond 3 days after surgery, even though the duration of the iSLN block can be further evaluated. Second, as the superior laryngeal nerve and the superior laryngeal artery are too small to locate, we injected the local anesthetic into the superior laryngeal nerve space, which did not depend on finding the superior laryngeal artery and superior laryngeal nerve. So the technique of US-guided iSLN block in our study was relatively simple with little stimulation, even though there is a risk of blocking the external branch of the superior laryngeal nerve and recurrent laryngeal nerve simultaneously. Thirdly, the use of ropivacaine, a kind of long-acting drug, tends to prolong the blocking block, which may increase the choking cough, aspiration and regurgitation after swallowing saliva. However, in our study, the patients in both groups did not suffer from choking cough, aspiration or regurgitation after swallowing saliva.

## Conclusions

US-guided iSLN block could be safely and effectively used for patients undergoing thoracic surgery with double-lumen bronchial intubation, and it could significantly decrease the postoperative incidence of sore throat.

## Data Availability

All data generated or analysed during this study are included in this published article.

## Abbreviations

ANOVA: Analysis of one-way variance
BIS: Bispectral index
DLTs: Double-lumen endobronchial tubes
ECG: Electrocardiogram
GA: General anesthesia
HR: Heart rate
i.v.: Intravenous
iSLN: Internal branch of the superior laryngeal nerve
NIBP: Non-invasive blood pressure
POST: Postoperative sore throat
SD: Standard deviations
SpO_2_: Oxygen saturation
US: Ultrasound
